# A Plea for Specialties

**Published:** 1869-01

**Authors:** Edward Rives


### A PLEA FOR SPECIALTIES.

BY PROF. EDWARD RIVES, M. D.

I have seen people die unnecessarily, under the most scientific and unexceptionable medication. There is then something more than a ministration to physical wants required. Clearly the association of mind and body is so intimate that the one may not be seriously diseased without an implication of the other. No one has a more profound respect for the wonderful revelations of physical and chemical sciences in connection with the profession of medicine than the writer, and no one can be more ready to recognize their value or avail himself of a knowledge of them. But with all the facilities which these sciences have afforded us in the various branches of the profession, these alone, it will be found, are not sufficient for the cure of disease. If man were a thing, certain causes would always produce certain effects upon him, just as definite relations exist between inorganic substances. But man is organic, and not only so, but of the most highly vitalized class; and more than all, is endowed with mind, that subtle incomprehensible link which connects us with the Creator, and this mind is so intimately blended with our physical nature that they mutually react upon each other, and we can not, with impunity, overlook the importance of moral influences in the treatment of disease. It is not at all infrequent in my experience, and, I suppose, in that of other physicians, who have watched closely the phenomena of disease, to see and mark sudden changes which can not be wisely attributed to medication or to physical infirmity. Such phenomena, if closely examined, may be referred to moral causes with a great degree of certainty. While I lay no claim to originality in bringing this subject before the profession. I can not shut my eyes to the fact that in actual practice we rely too much on the "*modus medendi*," and not enough on moral influences. We are too apt to refer every increase of perturbation to physical causes, every decrease to skillful medication. I trust I may not be misunderstood. It *is* absolutely necessary to know the pathology of disease and its appropriate medication, but this is not all. A thoughtless word or look, a want of attention to foolish whims, a want of tact on the part of attendants, a want of light or darkness, of silence or sound, and a hundred kindred little things which would scarcely impress a sane man, seriously disturb the mind of a sick one and react upon his physical nature. Such slight causes at the crisis of a grave disease—when life is suspended, as it were, by a thread—will often determine the result. I have known the change of a counterpane to have the happiest effect on a patient ill of typhoid fever who could not refer his mental disturbance to a definite cause. I have known the figures on the curtains of a sick room to be distorted into hideous images by the sick man. When these curtains were removed, he sank into a gentle slumber, and awoke on the ascending scale of health, sight, hearing, taste, smell, feelings, whims, all, must be made conducive to recovery, else they retard or prevent it. The mental wants of a sick man differ from those of a well one as widely as do his physical wants, these must be as wisely administered to in the former as the latter case. Alas, how few of us are qualified to soothe the weary brain distorted with sickly imaginations. We can not assume such qualities, for the sick man's penetration concerning his personal welfare is often keener than when in a state of health. He observes in silence but with watchful eyes every movement of his attendants, every look, every whisper, and his imagination may distort the kindest motives into inattention or indifference. How often do we see extreme officiousness endeavoring to take the place of intelligent active sympathy, all with the best intentions, but bearing the worst consequences. Adaptability to the sick room can not be taught, it is the essence without which all knowledge will be in vain for purposes of cure. It can not be learned, for it is born with the individual and is not an acquirement, it *is* the physician. This instinctive sympathy, this electrical unison between the minds of the physician and patient is a gift without which not one can hope to be successful in the sick room. All of us may learn to minister to the physical nature in accordance with scientific facts. Few of us combine this knowledge with the intuitive perception of the state of the patient's mind. Without such combination the patient may get well in spite of you, but not because of you. It may be a startling opinion to state that all who have a knowledge—in the usual acceptation of the term—of the profession are not competent to cure disease, are not fitted for the sick room. Superadded to knowledge he must have the genius, talent, or whatever you choose to call it, to which I have referred, of identifying himself with the mental condition of the patient. A close observation of the troublesome vagaries, whims and caprices of the sick, and of the apparently little nothings which often dispel them, will convince the candid mind that these little nothings are really often of vital importance, and require an extraordinary perception to discover. How are we to know of this peculiar adaptation to the sick room, is a question very pertinent, but I confess, difficult to answer. Ones vanity may mislead him, but we would leave the decision to each individual for himself and those who are laboring for the development of truth will not deceive themselves. What shall we do for a living? is an equally pertinent question, but one far easier to answer than the first. To cure disease is but one out of many branches of the profession. The very general aspiration to become physicians, I mean curers of the sick, retard the progress of our science. How few workers have we comparatively in the unexplored field of pathology, physiology, chemistry, microscopy and kindred sciences, each one of which surely embrace enough to occupy limitless generations, and if properly pursued as a means of livelihood would be quite as remunerative, and perhaps more so, than the general practice. The how and why I leave to the intelligence of the reader, contenting myself with the assertion that no one has yet attained excellence in these pursuits without corresponding emolument in money and reputation.

I take the ground unhesitatingly that, in general, it is a physical and intellectual impossibility for one man to embrace the details of all the branches of medicine. It is true that a knowledge of their general principles is not only attainable but *absolutely necessary* to all workers in the profession, but we must work in detail if we expect to advance. We have had too much generalization, and until a comparatively recent date, the study of specialties has, by some absurd species of reasoning, been connected with quackery One of the chief charms, it has often struck me, in the profession, is that it furnishes an appropriate nidus, so to speak, for every class of mind to develope. All have room in some specialty. In the choice, however, of a specialty, we are too apt to be led to that which has proved remunerative to others, though we may not have any special inclination or adaptation to it. That specialty will be the most remunerative which is most in consonance with the character of our minds. Each class of minds should work in different spheres. That class which can collect, arrange, harmonize facts, and make practical application of the grand total, is comparatively few in number, and by reason of this harmonizing faculty, is composed of men who, in my estimation, are alone to be entrusted with the general sick, who have that mysterious influence over, and sympathy with the sick mind so necessary in the cure of disease. At first contemplation of this view of the subject, the specialist may be startled, nay, indignant, at the seemingly inferior position to which I have assigned him, but aspiring myself to a specialty, I do not regard the subject viewed in this light with any thing but satisfaction. If the specialist is not fitted for general practice, neither is the general practitioner adapted to the work of the specialist. They are each necessary to the other. Each equally necessary to the world, and each, if faithful to his duty, will be amply remunerated. The sum of all—there are too many physicians, too few specialists. Am I right?

